# Supplementary material for: Drug repurposing for aging research using model organisms
Source: Aging Cell. 2017 Jun 16;16(5):1006–15. doi: 10.1111/acel.12626 (PMC5595691; doi:10.1111/acel.12626)
Supplement: Supplementary file 7 — Data S1 Zip‐Archive of all report cards. [file ACEL-16-1006-s007.zip › RC_PIT.pdf]

PIT

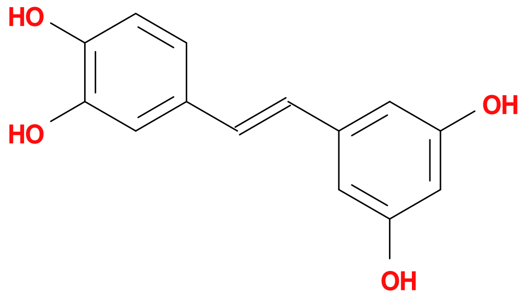

Database identifiers

ChEMBLCompound	CHEMBL69863
DrugBank	DB08399
CHEBI	28814
ZINC	ZINC00014036
eMolecules	593143

Ranking

	Rank	Score
Drosophila	436/697	0.33
C. elegans	NA	NA

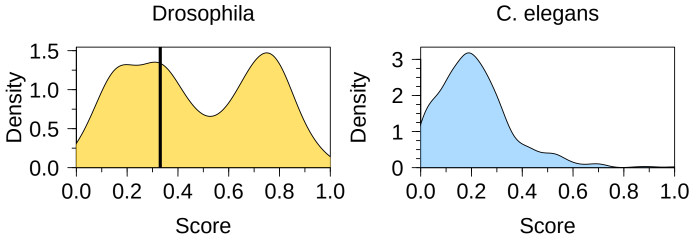

	Ageing implication	Domain conservation	Binding site conservation	Binding affinity	Bioavailability	Lipinski	Promiscuity	Purchasability	Drug approval	**Total**
Drosophila	1.0	0.687	0.791	0.319	(0.9)	0.0	-0.0	0.1	0.075	0.33
C. elegans	NA	NA	NA	NA	NA	NA	NA	NA	NA	NA

Names

- **piceatannol**
- 3,3',4'5-Tetrahydroxystilbene
- 3-hydroxyresveratol
- 4-(E)-2-(3,5-dihydroxyphenyl)vinylbenzene-1,2-diol

Roles

ChEBI entry 28814 has no roles

Status

Approved drug (according to ChEMBL)	No
Classification (according to DrugBank)	experimental
Number of Rule of 5 violations	0
Binding affinity to original target in log units (RF-Score prediction)	4.24
Burns *C. elegans* bioavailability prediction	0.91

Compound Target Characteristics

NAD-dependent protein deacetylase sirtuin-3, mitochondrial

Best gene implication in ageing for this target family came from gene Q9I7I7 annotated in UniProt release 2014_02. Annotation GO 8340 (determination of adult lifespan) was Inferred from Mutant Phenotype

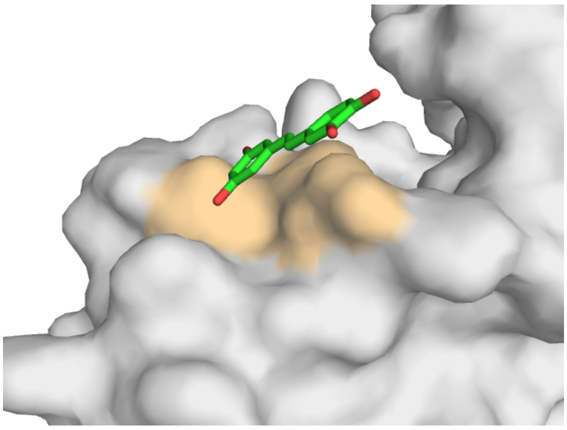

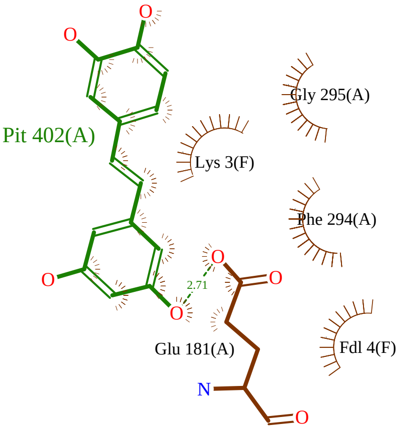

protein	amino acids contacts (binding site)
PDB:4hd8:chainA:Q9NTG7	E F G
tr:E9PK80:E9PK80_HUMAN	E F G
tr:E9PN58:E9PN58_HUMAN	E F G
sp:Q9NTG7:SIR3_HUMAN	E F G
tr:B2RZ31:B2RZ31_RAT	E F G
tr:C6ZII9:C6ZII9_RAT	E F G
tr:Q4FJK3:Q4FJK3_MOUSE	E F G
tr:D3YTK6:D3YTK6_MOUSE	E F G
sp:Q8R104:SIR3_MOUSE	E F G
sp:Q9I7I7:SIRT2_DROME	D F G
sp:P53686:HST2_YEAST	D F G

	whole protein		domain-based		contact-based	
protein	ident	simil	ident	simil	ident	simil
PDB:4hd8:chainA:Q9NTG7	1.0	1.0	1.0	1.0	1.0	1.0
tr:E9PK80:E9PK80_HUMAN	0.8	0.8	1.0	1.0	1.0	1.0
tr:E9PN58:E9PN58_HUMAN	0.78	0.79	0.93	0.93	1.0	1.0
sp:Q9NTG7:SIR3_HUMAN	1.0	1.0	1.0	1.0	1.0	1.0
tr:B2RZ31:B2RZ31_RAT	0.55	0.62	0.85	0.96	1.0	1.0
tr:C6ZII9:C6ZII9_RAT	0.63	0.74	0.85	0.96	1.0	1.0
tr:Q4FJK3:Q4FJK3_MOUSE	0.55	0.62	0.85	0.96	1.0	1.0
tr:D3YTK6:D3YTK6_MOUSE	0.48	0.55	0.61	0.67	1.0	1.0
sp:Q8R104:SIR3_MOUSE	0.61	0.72	0.85	0.96	1.0	1.0
sp:Q9I7I7:SIRT2_DROME	0.34	0.55	0.43	0.68	0.67	0.79
sp:P53686:HST2_YEAST	0.26	0.49	0.34	0.64	0.67	0.79

Sirt2 (FBgn0038788) associated phenotypes

heat sensitive, lethal - all die before end of pupal stage, some die during pupal stage

(Information from FlyBase)

Sirt2 (UniProt:Q9I7I7) annotation

Function: NAD-dependent protein deacetylase (By similarity). May be involved in the regulation of life span. (, PubMed:17159295).
Cofactor: Zn(2+)Note=Binds 1 zinc ion per subunit. ;
Disruption phenotype: Causes lethality during development. Induced silencing shortens life span. (PubMed:17159295).

(Information from UniProt)
